# T-cell dysregulation is associated with disease severity in Parkinson’s Disease

**DOI:** 10.1186/s12974-021-02296-8

**Published:** 2021-10-30

**Authors:** Divisha Bhatia, Veselin Grozdanov, Wolfgang P. Ruf, Jan Kassubek, Albert C. Ludolph, Jochen H. Weishaupt, Karin M. Danzer

**Affiliations:** 1 Neurology, University Clinic, University of Ulm, Albert-Einstein-Allee 11, 89081 Ulm, Germany; 2 German Center for Neurodegenerative Diseases (DZNE), Ulm, Germany; 3 Division for Neurodegenerative Diseases, Neurology Department, University Medicine Mannheim, Heidelberg University, Mannheim, Germany

**Keywords:** Parkinson’s Disease, T cells, Disease severity, PHA, Gene expression

## Abstract

The dysregulation of peripheral immunity in Parkinson’s Disease (PD) includes changes in both the relative numbers and gene expression of T cells. The presence of peripheral T-cell abnormalities in PD is well-documented, but less is known about their association to clinical parameters, such as age, age of onset, progression rate or severity of the disease. We took a detailed look at T-cell numbers, gene expression and activation in cross-sectional cohorts of PD patients and age-matched healthy controls by means of flow cytometry and NanoString gene expression assay. We show that the well-pronounced decrease in relative T-cell numbers in PD blood is mostly driven by a decrease of CD8^+^ cytotoxic T cells and is primarily associated with the severity of the disease. In addition, we demonstrate that the expression of inflammatory genes in T cells from PD patients is also associated with disease severity. PD T cells presented with increased activation upon stimulation with phytohemagglutinin that also correlated with disease severity. In summary, our data suggest that the consequences of disease severity account for the changes in PD T cells, rather than age, age of onset, duration or the disease progression rate.

## Introduction

Neuroinflammation in Parkinson’s Disease (PD) includes the infiltration of T cells into the diseased CNS, with reactive T cells surrounding neurons and likely contributing to their demise [1]. Neuroinflammation in the CNS is complemented by well-described immune changes in the periphery, including changes in the absolute and relative numbers of monocytes and lymphocytes and their subsets [2–5], increased activation by inflammatory stimuli [2, 6] and the presence of auto-immune components, such as T cells and antibodies reactive to alpha-synuclein [7, 8]. Recently, T cells have received special attention due to the discovery that the genetic association of HLA-DR haplotypes with PD [9, 10] underlies an increase of T cells reactive to a-synuclein [8, 11]. Other phenotypic changes of T cells in PD are also known: reduced absolute lymphocyte counts, decreased absolute and relative counts of total T cells, decreased absolute and relative counts of CD4 + , and sometimes also CD8 + lymphocytes, increased Th1/Th2 and Th17/Treg ratios and increased expression of inflammatory cytokines [5, 12–27]. However, most of these changes are also found during healthy aging, making it difficult to discern the impact of a disease, such as PD, which presents with a very broad range of onset (~ 30–90 years) and variable progression rate. Most studies so far concentrated on disease severity, or disease duration as a proxy of it, without considering the influence of sex and aging. As T-cell characteristics are an easily accessible parameter which can be tracked during the course of disease-modifying therapies, it is important to better understand which features correlate with disease severity and how they are biased by other clinical parameters, such as sex, age, age at onset and progression rate of the disease. Therefore, we investigated several T-cell features in cross-sectional cohorts of PD patients and healthy controls (HC): T-cell numbers as a robust and universal, well-described alteration in PD, T-cells activation and expression of inflammatory genes.

## Methods

### Study participants

All experimental work with human samples was carried out in agreement with the Declaration of Helsinki and under the approval of the Ethics Committee of the University of Ulm, Germany (“Antrag Nr. 20/10”). All study participants provided informed written consent to participate in the study. PD patients and healthy controls were recruited through the Universitäts- und Rehabilitationskliniken Ulm (RKU). Age-matched healthy controls were recruited based on negative history of neurological disease. PD patients and healthy controls with a history of acute or chronic inflammatory or autoimmune disease or medication were excluded from the study. Disease severity was assessed by an experienced neurologist according to the Hoehn & Yahr (HY) PD staging criteria [28]. Demographic and clinical characteristics including medication and selected comorbidities are summarized in Table 1.

**Table 1:** Clinical, demographic and T-cell characteristics of the experimental cohorts

	*n*	Age	Sex (f/m)	Age at diagnosis	Disease duration	Progression rate	Disease severity	T-cells		
								% CD3 +	% CD4 +	% CD8 +
Immunophenotyping (Figs. 1, 2)	36	Information available: *n* = 36/36						Information available: *n* = 36/36		
Mean ± stdev		70.3 ± 9.5	12/24	60.0 ± 9.8	10.3 ± 6.0	0.33 ± 0.32	2.4 ± 0.94	11.5 ± 5.4	7.8 ± 3.4	2.4 ± 2.5
Median (interq. range)		72.1 (63.8–78.1)		62.5 (52.8–66.0)	8.7 (7.0–14.8)	0.25 (0.20–0.40)	2.0 (1.0–3.0)	9.8 (8.6–13.2)	6.7 (6.0–8.4)	1.8 (1.1–2.9)
Gene expession (Fig. 3)	16	Information available: *n* = 16/16						Information available: *n* = 10/16		
Mean ± stdev		68.1 ± 9.6	4/12	58.7 ± 11.2	9.4 ± 7.5	0.44 ± 0.58	2.3 ± 0.99	14.7 ± 7.6	9.4 ± 4.5	3.9 ± 4.1
Median (interq. range)		70.5 (64.0–74.0)		59.0 (47.8–67.8)	7.0 (5.0–11.0)	0.22 (0.19–0.50)	2.0 (1.5–2.6)	10.9 (9.3–19.5)	6.7 (6.4–12.1)	2.8 (1.7–4.1)
T-cell function (Fig. 4)	23	Information available: *n* = 23/23						Information available: *n* = 0/23		
Mean ± stdev		68.9 ± 10.5	6/17	60.2 ± 9.2	8.7 ± 6.2	0.51 ± 0.46	2.4 ± 1.0	–		
Median (interq. range)		67.0 (63.5–78.5)		60.0 (51.5–69.0)	8.0 (3.0–13.5)	0.31 (0.24–0.83)	2.0 (2.0–3.0)			

	*n*	Medication						Comorbidities			
		No medication	Levodopa	Dopamine Agonists	COMT Inhibitors	MAO-B Inhibitors	DOPA-decarboxylase Inhibitors	No comorbidities	Dementia	Hallucinations	Other comorbidities
Immunophenotyping (Figs. 1, 2)	36	Information available: *n* = 32/36						Information available: *n* = 33/36			
*n*		0/32	28/32	22/32	18/32	14/32	28/32	3/33	1/33	5/33	30/33
Gene expression (Fig. 3)	16	Information available: *n* = 13/16						Information available: *n* = 13/16			
*n*		0/13	12/13	9/13	6/13	4/13	12/13	2/13	1/13	3/13	11/13
T-cell function (Fig. 4)	23	Information available: *n* = 23/23						Information available: *n* = 0/23			
*n*		0/23	14/23	12/23	8/23	13/23	14/23	–			

Continuous variables presented as mean ± standard deviation *(“stdev”)* and median with interquartile range *(“interq. range”, 1. and 3. quartile)*. Side effects from medication are not included in comorbidities. Comorbidities listed under *“Others”*: *Anxiety disorder, atherosclerosis, bursitis, Carpal tunnel syndrome, cholelithiasis, chronic kidney failure, chronic venous insufficiency, reactive collagenosis, convex scoliosis, COPD, coronary bypass, coronary heart disease, Crohn’s disease, degenerative joint disease, degenerative spine disease, depression, diplopia, dorsal funiculus symptoms, heart arrythmia with atrial fibrillation, heart valve fibrosis, heart valve sclerosis, hemodynamic orthostatic vertigo, hypercholesterolemia, hyperlipoproteinemia, hyperopia, hyperparathyroidism, hypertension, hyperuricemia, hypothyroidism, inclusion body myositis, lactose intolerance, lentigo maligna, lumbago, mitral insufficiency, myalgia, myopathy, nephrolithiasis, osteochondrosis, osteoporosis, chronic pain syndrome, polyarthritis, prostatic adenoma, sensorymotor polyneuropathy, sleep apnea, spinal disc herniation, supraventricular extrasystole, tachycardia, thrombophlebitis, tinnitus, type 2 diabetes, vitamin B12 deficiency*

*COMT catechol-O-methyltransferase, MAO-B Monoamine oxidase B, DOPA 3,4-dihydroxyphenylalanine*

### Flow cytometry

Peripheral blood samples were collected with Li-Heparin Monovettes (Sarstedt) and processed within 3 h. Red blood cells were eliminated by osmotic lysis with RBC lysis buffer as described before [2]. Leucocytes were then washed, re-suspended in FACS buffer (DPBS + 10% FCS). Unspecific binding of antibodies to cell surface receptors was blocked with Fc Block (1:20, BioLegend) or human TruStain Fc Block (BioLegend) for 20 min at 4 °C. Leucocytes were stained with antibodies specific for CD3 (1:50, BioLegend; UCHT1-PB), CD4 (1:100, BioLegend RPA-T4-PE), CD8 (1:100, BioLegend, SK1-APC), and CD69 (1:50, BioLegend; FN50-FITC) for 25 min at 4 °C in the dark. After washing, the stained cells were fixed for 20 min in 2% PFA, filtered through a 70 µM cell-strainer and characterized by flow cytometry on a LSRII flow cytometer (BD) using a combination of unstained cells, internal negative population controls and single-color staining for color compensation. Therefore, isotype-matched, host-matched monoclonal antibodies were used as negative control: mouse IgG2a, κ (BioLegend; MOPC-173-PE-Cy7), mouse IgG1, κ (BioLegend; MPOK-21-PB), mouse IgG1, κ (BioLegend; MOPC-21-PE), mouse IgG2a, κ (eBioscience; P3.6.2.81-APC). Flow cytometry data was processed with FACS Diva™ software (BD).

### T-cell culture

Blood samples were obtained by venipuncture and collected with K3-EDTA Monovettes (Sarstedt). Peripheral blood mononuclear cells (PBMCs) were enriched by density gradient centrifugation over Histopaque^®^-1077 (Sigma-Aldrich) as previously described [29]. CD3^+^ T cells were enriched by magnetic-activated cell selection with magnetic-beads-coupled monoclonal antibodies to CD3 (Miltenyi Biotec). Purified CD3^+^ T cells were cultured in RPMI1640 cell culture medium (Gibco) supplemented with 10% FCS (PAA) and 1% penicillin/streptomycin (PAA) at 37 °C, 5% CO_2_. The purity of the isolated T cells was controlled by flow cytometry. For stimulation of T cells, cells were pelleted at 500 g for 5 min and re-suspended in T-cell activation media (Gibco; PB-MAX™ Karyotyping Medium) at a total density of 4 × 10^5^ cells/mL. After 72 h of stimulation, the activation of the T cells was assayed by flow cytometry for CD69 expression.

### Inflammatory gene expression

Expression of inflammatory genes was quantified on with the NanoString Inflammation Panel on an nCounter^®^ analyzer with standard parameters according to the supplier’s instructions. Total RNA was isolated from T cells purified by CD3 + magnetic selection as described above. Gene expression data was normalized to the geomean expression of six house-keeping genes and differential expression analyzed with DESeq2 [30].

RT-qPCR was performed as described before [2] with total mRNA isolated from purified CD3^+^ T cells. Gene expression of CXCL3 (5′-CCT GCC CTT ACC AGA GCT GAA A-3′ and 5′-ATT AAG TCC TTT CCA GCT GTC CC-3′), CCR1 (5’-ATG CAA CTC CGT GCC AGA AGG-3′ and 5′-AGG TCA GAA ATG GCC AGG TTC A-3′) and CXCL2 (5′-CCA CTG TGA TAG AGG CTG AGG AA-3′ and 5′-ATA CAT TTC CCT GCC GTC ACA TT-3′) was quantified with the 2^−ΔΔCt^ method [31] relative to the expression of B2M (5′-AGA TGA GTA TGC CTG CCG TG-3′ and 5′-GCG GCA TCT TCA AAC CTC CA-3′), TBP (5′-CCC ATG ACT CCC ATG ACC-3′ and 5′-TTT ACA ACC AAG ATT CAC TGT GG-3′) and RNU6 (5′-CTC GCT TCG GCA GCA CAT-3′ and 5’-AAC GCT TCA CGA ATT TGC GT-3′). The expression of each reference gene was controlled against the expression of the other two reference genes to exclude co-regulation of the reference and inflammatory genes. Genomic DNA and PCR artifacts were excluded with NTC and NRT controls and by optimization of primer design.

### Data analysis

Statistical analyses were carried out with R 4.0.3 (R Core Team). Gaussian distribution was tested with D’Agostino and Pearson omnibus normality test, Shapiro–Wilk normality test and Kolmogorov–Smirnov normality test. Statistical significance between distributions of two groups was tested with Mann–Whitney *U* test; correlation with Spearman’s correlation and odd ratios with Chi-square test and Fisher’s exact test. Multiple testing was controlled with Bonferroni. Interaction of covariates on dependable variables was tested with two-way ANOVA. Density bandwidth estimation for the stratification of PD patients according to clinical parameters was performed with the Sheather and Jones method to avoid under- and oversmoothing [32]. Hierarchical clustering of gene expression data was performed with average linkage and Euclidean distance. Differential gene expression was analyzed with DESeq2 with standard parameters [30]. Statistical significance of differential expression data was corrected for multiple testing with FDR [33]. Weighted gene co-expression network analysis was performed with the WGCNA R package [34, 35]. Flow cytometry data were analyzed with FACS Diva 8.0.1 software (BD).

## Results

### Decrease of total CD3^+^ T cells in PD is associated with disease severity

**Fig. 1:**
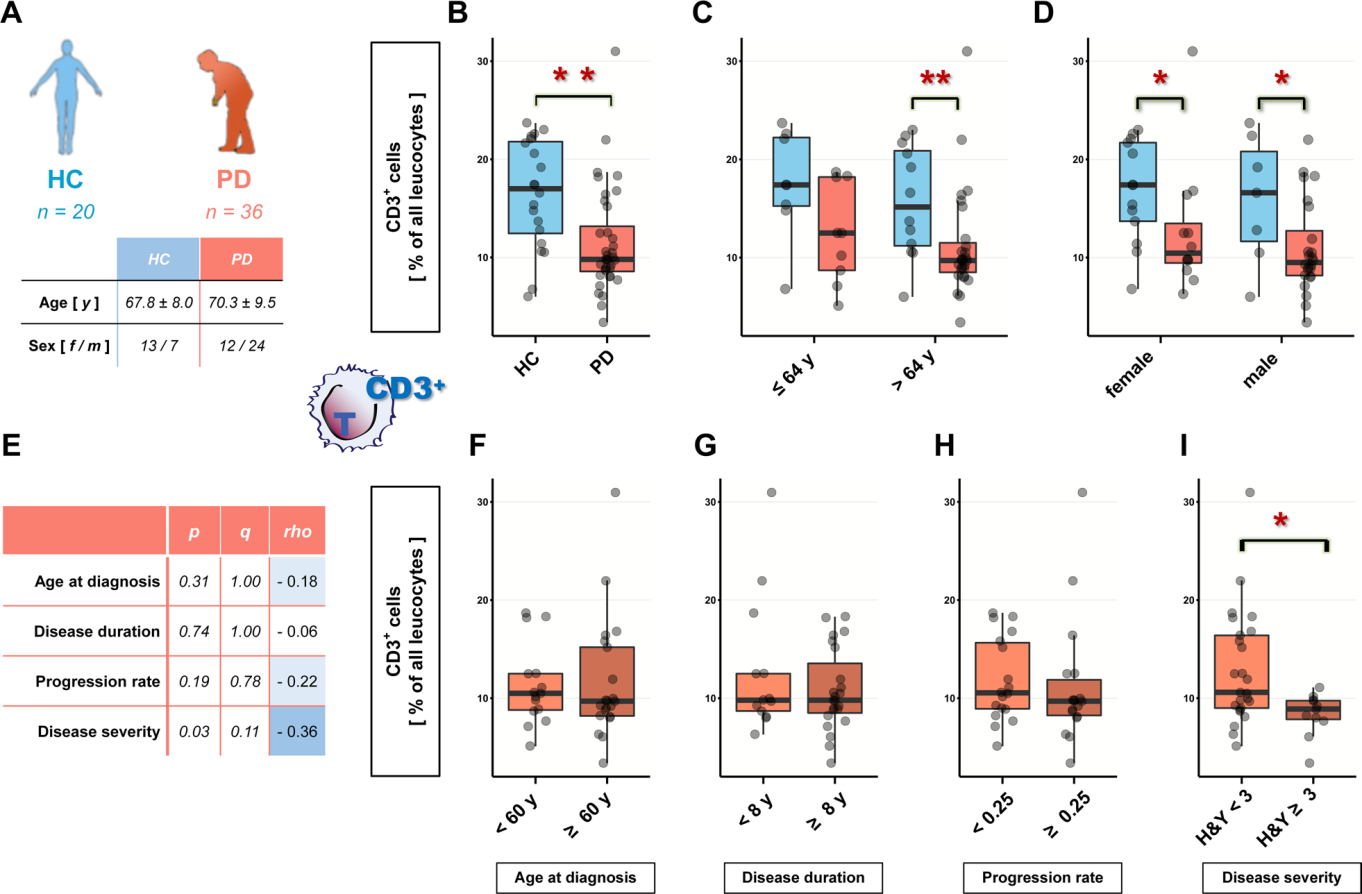
Decrease of total CD3^+^ T cells in PD is associated with disease progression. **A**, **B** Flow-cytometric analysis of peripheral blood leucocytes from HC *(n* = 20, f/m = 13/7) and PD patients *(n* = 36, 12/24 f/m) reveals a significant decrease of total CD3^+^ T cells (as % of all leucocytes) in PD. **C**, **D** T cells are decreased in PD in both age groups (≤ 64 years and > 64 years) and in both sexes (two-way ANOVA: case **p < *0.*01, sex *p* > *0.*36, age *p* > *0.35*, no statistically significant interactions). **E** T-cell decrease correlates significantly with disease progression (*Spearman’s* ρ). **F–I** CD3^+^ T cells are significantly decreased in PD patients with advanced disease progression (H&Y ≥ 3) **(I)**, but not earlier age at onset **(F)**, longer disease duration **(G)**, or higher progression rate (**H,** H&Y/year). Boxplots: median ± interquartile range; *p < 0.05, **p < 0.01, Mann–Whitney *U* test, two-way ANOVA

To investigate how clinical parameters of PD relate to the changes observed in the numbers of T cells in PD, we investigated the relative abundance of total CD3^+^ T cells, as well as the subpopulations of CD3^+^CD4^+^ and CD3^+^CD8^+^ T cells in a cross-sectional cohort of 36 PD patients (mean age 70.3 ± 9.5; f/m = 12/24; mean H&Y 2.4 ± 0.94) and 20 age-matched (*p* > *0.32*) healthy controls (mean age 67.8 ± 8.0; f/m = 13/7) (Fig. 1A). We observed a statistically significant decrease of CD3^+^ T cells (as % of all leucocytes) in PD patients (Fig. 1B), which was independent of differences in the age (Fig. 1C, two-way ANOVA **p < *0.01* for HC vs. PD, *p* > *0.35* for ≤ 64 years vs. > 64 years, *p* > *0.86* for interaction) or sex of PD patients and healthy controls (Fig. 1D, two-way ANOVA **p < *0.01* for HC vs. PD, *p* > *0.36* for f vs. m, *p* > *0.78* for interaction). We then investigated the association of CD3^+^ cell decrease with age at initial diagnosis, disease duration, disease progression rate and disease severity assessed by H&Y score. Notably, only disease severity correlated with the observed decrease of CD3^+^ T cells (Fig. 1E, *p < *0.05, q* < *0.16, Spearman’s ρ* = − *0.36*). Stratification by clinical parameters revealed a statistically significant difference in CD3^+^ T cells numbers between patients with relatively earlier disease (H&Y < 3) and relatively advanced disease (H&Y ≥ 3) (Fig. 1I, *p < *0.05*), but not between PD patients stratified by age at diagnosis (< 60 years vs. ≥ 60 years, Fig. 1F), disease duration since initial diagnosis (< 8 years vs. ≥ 8 years) and disease progression rate (assessed by H&Y stage increase per year since initial diagnosis, < 0.25 vs. ≥ 0.25, Fig. 1G). Furthermore, a multivariate analysis of variation with the discretely stratified clinical traits confirmed that only disease severity has a significant effect on the number of CD3^+^ T cells, with no detectable interaction between any combination of the clinical traits.

**Fig. 2:**
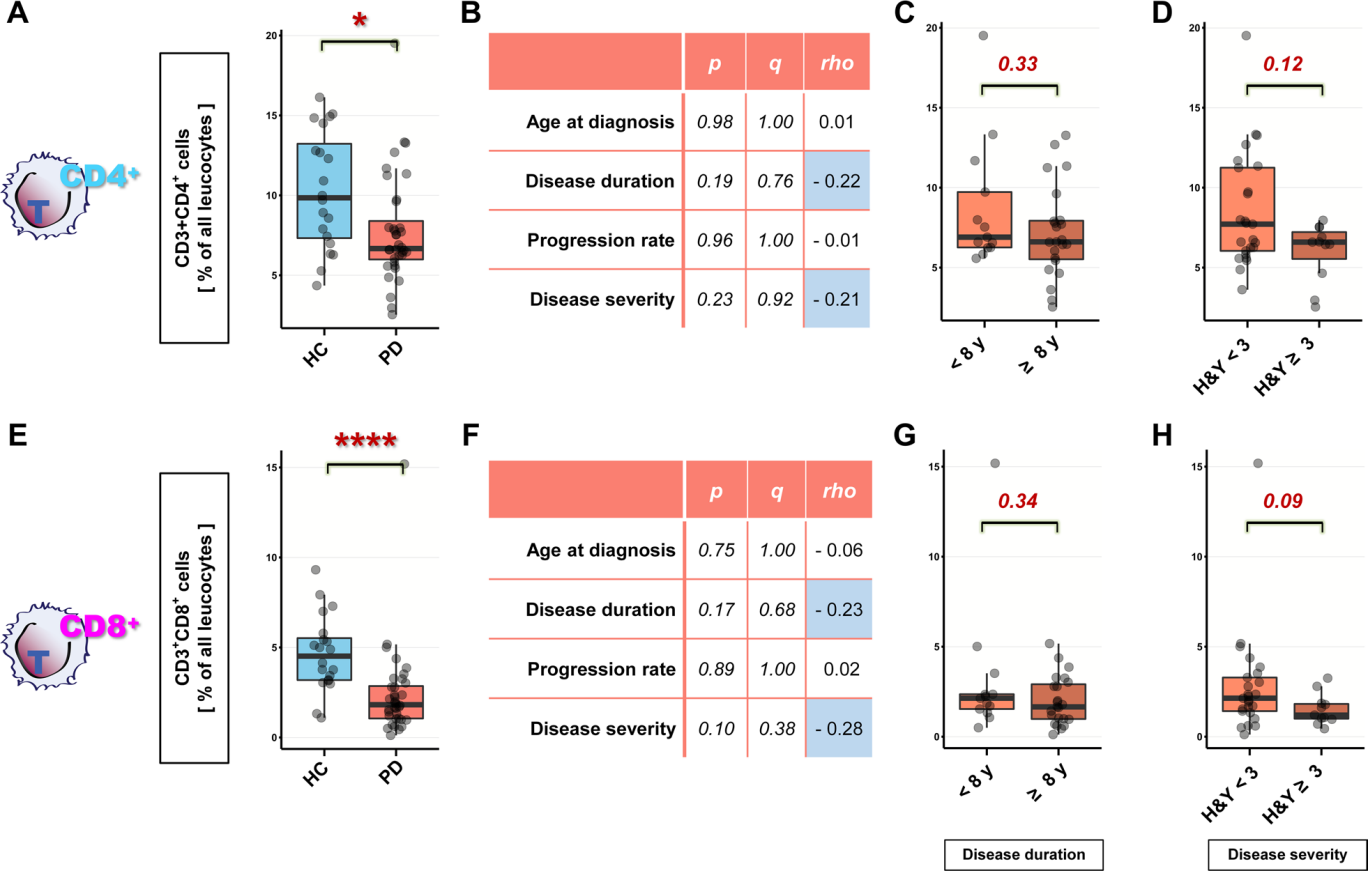
CD4^+^ and CD8^+^ T cells are decreased in PD. **A** CD3^+^CD4^+^ T cells are significantly decreased in PD patients (as % of all leucocytes, two-way ANOVA: case *p < *0.05*, sex *p* > *0.36*, age *p* > *0.41*, no statistically significant interactions). **B** Correlation of CD3^+^CD4^+^ cell numbers with PD clinical traits (*Spearman’s ρ*). **C**, **D** CD3^+^CD4^+^ cell numbers in PD patients stratified by disease duration (**C**) and disease progression (**D**). **E** CD3^+^CD8^+^ T cells are significantly decreased in PD patients (as % of all leucocytes, two-way ANOVA: case **p < *0.01*, sex *p* > *0.41* age *p* > *0.62*, no statistically significant interactions). **F** Correlation of CD3^+^CD8^+^ cell numbers with PD clinical traits (*Spearman’s ρ*). **G**, **H** CD3^+^CD8^+^ cell numbers in PD patients stratified by disease duration (***G***) and disease progression (**H**). Boxplots: median ± interquartile range; *p < 0.05, ****p < 0.0001, Mann–Whitney *U* test, two-way ANOVA

Next, we investigated whether the decrease of CD3^+^ T cells in PD patients results from changes in the abundance of the T-cell subpopulations CD3^+^CD4^+^ or CD3^+^CD8^+^ T cells, and whether the abundance of these cell types also associates with clinical parameters of the disease. We found that both CD3^+^CD4^+^ and CD3^+^CD8^+^ T cells were significantly decreased in PD patients (Fig. 2A, E). However, CD3^+^CD8^+^ T cells showed a stronger decrease than CD3^+^CD4^+^ T cells (fold change of medians CD4^+^: 0.68, CD8^+^: 0.40), resulting in an increase of the *relative* percentage of CD3^+^CD4^+^ T cells and the CD4^+^:CD8^+^ cell ratio in PD patients. The decrease of each cell type alone was not significantly associated with any clinical trait, but showed a trend for association with disease severity (Fig. 2B–D, F–H; *p* < *0.13* (CD3^+^CD4^+^), *p* < *0.09* (CD3^+^CD8^+^)). We found no significant correlation of the absolute or relative numbers of CD4 + and CD8 + T cells with any clinical parameter of PD.

### Expression of inflammatory genes and activation by PHA in T cells from PD patients are associated with disease severity

**Fig. 3:**
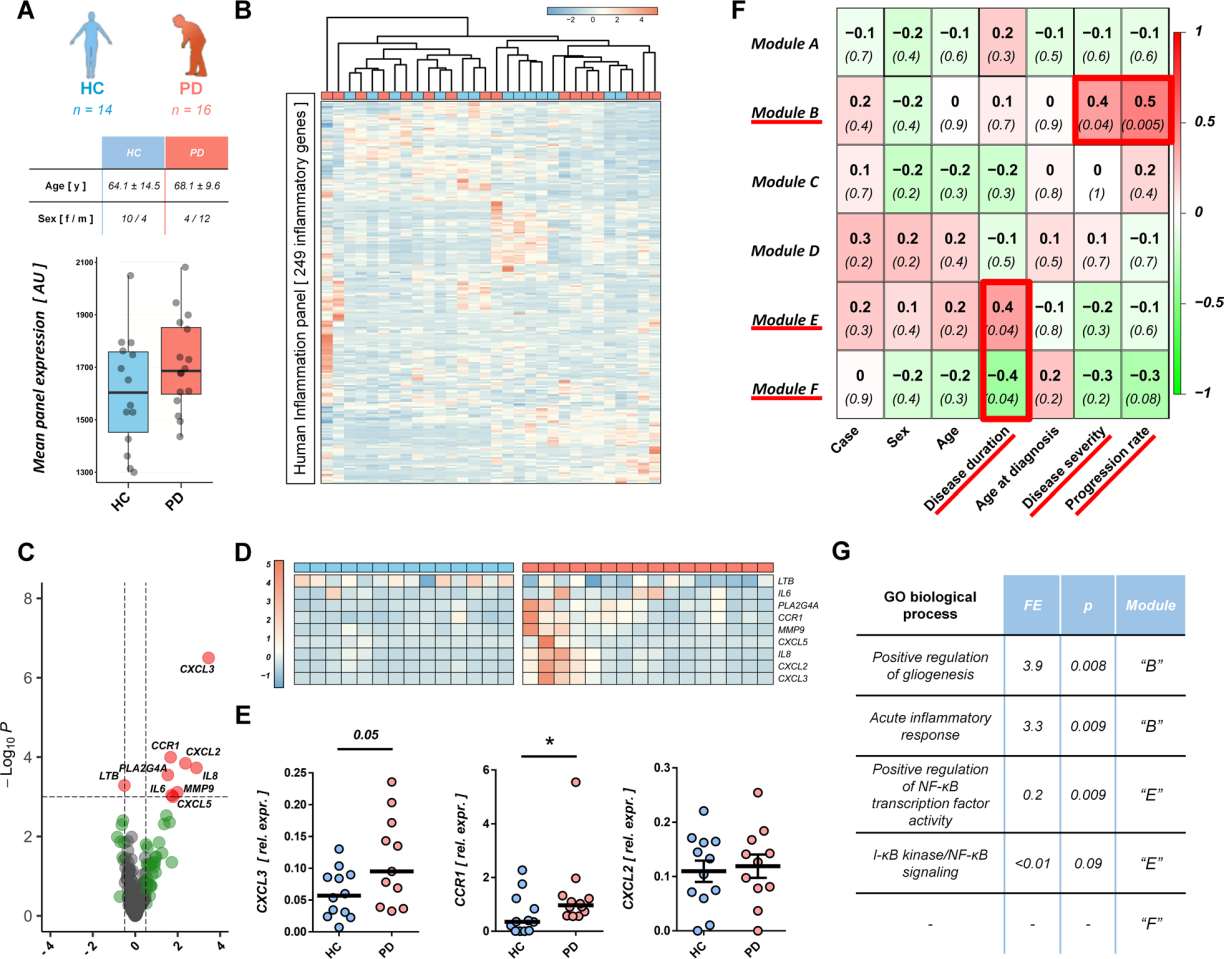
Expression of inflammatory genes in T cells from PD patients is associated with disease progression and disease duration. **A** Expression of 249 inflammatory genes was quantified with a NanoString expression assay in isolated CD3^+^ T cells from HC (*n* = 14, f/m = 10/4) and PD patients (*n* = 16, f/m = 4/12). Mean expression of all 249 quantified transcripts was slightly increased in PD patients (*p* = 0.24). **B** PD patients could not be identified based on the expression signature of all 249 transcripts (complete linkage with Euclidean distance). **C** Volcano plot and heatmap (**D**) of the differential expression analysis between HC and PD T cells. Nine genes were significantly differentially expressed in PD T cells (fdr* q* < 0.05, fold change < − 1.4/> 1.4). None of the nine genes’ expression was significantly affected by the age or sex of participants (two-way ANOVA). **E** Significant up-regulation of selected transcripts could be verified in an independent validation cohort by RT-qPCR (HC/PD* n* = 12/12). **F** WGCNA analysis reveals six blocks (modules “A”–“F”) of genes, which are co-expressed in a similar fashion amongst all samples. The weighted co-expression scores of these modules do not correlate significantly with case (HC or PD), sex, age or age at disease onset. Three gene modules (“B”, “E” and “F”) significantly correlated with disease duration and disease progression in PD patients. **G** GO enrichment analyses of modules “B”, “E” and “F” against the full list of all 249 quantified inflammatory genes reveals association of disease progression with genes important in acute inflammatory response and gliogenesis, and of disease duration with I-κB/NF-κB signaling. Boxplot: median ± interquartile range; lines (**E**): median; heatmap rows (genes) scaled to *z*-scores; *p < 0.05, Mann–Whitney *U* test; WGCNA correlation assessed with Pearson’s correlation coefficient; GO fdr *q* < 0.05

The association of inflammation with PD progression is well-documented. Therefore, to further characterize the dysregulation of T cells in PD patients and its association to clinical parameters of the disease, we purified CD3^+^ T cells by magnetic-activated cell sorting in a cross-sectional cohort of PD patients (*n* = 16, mean age 68.1 ± 9.6; f/m = 4/12) and healthy controls (*n* = 14, mean age 64.1 ± 14.5; f/m = 10/4) and quantified the expression of 249 inflammatory genes with the NanoString Inflammation panel expression assay (Fig. 3A). We observed that the total expression of all 249 inflammatory genes was slightly, but not statistically significantly increased in PD patients (Fig. 3A, *p* < *0.25*). Unsupervised hierarchical clustering demonstrated that the expression levels of all 249 quantified inflammatory genes did not robustly differentiate between PD patients and healthy controls, although some sub-clusters were visible (Fig. 3B). Differential expression analysis identified one gene which was significantly down-regulated and eight genes which were significantly up-regulated in PD T cells (Fig. 3C; fc < / > 0.5, *q* < *0.05*), with an enrichment of cytokines and chemokines (IL6, IL8, CXCL2, CXCL3, CXCL5). Due to the uneven distribution of male and female volunteers between the PD patients and healthy controls groups, we controlled the expression of all nine differentially expressed genes for an effect of sex or age of the volunteers with multivariate two-way ANOVA. None of the nine differential genes’ expression was affected by sex or age of the study participants and no interaction between case and age/sex was observed at *p* < *0.05*. The expression of these genes was highly variable in the PD patients group (Fig. 3D). Furthermore, we could validate the differential expression of two of the three top differentially expressed genes in an independent validation cohort by RT-qPCR (Fig. 3E, HC/PD *n* = 12/12; HC: mean age 64.7 ± 7.9, f/m = 5/7; PD: mean age 69.1 ± 5.8, f/m = 5/7, mean disease severity: 2.2 ± 0.9 HY, mean disease duration: 7.5 ± 5.4 years). Next, to investigate whether PD clinical traits are associated with the expression of inflammatory genes in T cells, we performed a weighted gene co-expression network analysis (WGCNA), clustering the 249 inflammatory genes into six modules of similar expression pattern across all PD patients and healthy controls (“Modules A–F”). Correlation of the module expression scores with clinical parameters revealed that the expression of two modules (“E” and “F”) was associated with disease duration, while the expression of one module (“B”) was significantly, positively correlated to disease severity and progression rate (Fig. 3F). To gain further mechanistic insight, we analyzed the functional enrichment of Gene Ontology (GO) terms of the genes in modules “B”, “E” and “F” against the full list of 249 quantified inflammatory genes (Fig. 3G). We found that genes involved in the regulation of gliogenesis and acute inflammatory response were significantly enriched (**p < *0.01, q* > *0.05*) in module “B”, which was associated with disease severity.

**Fig. 4:**
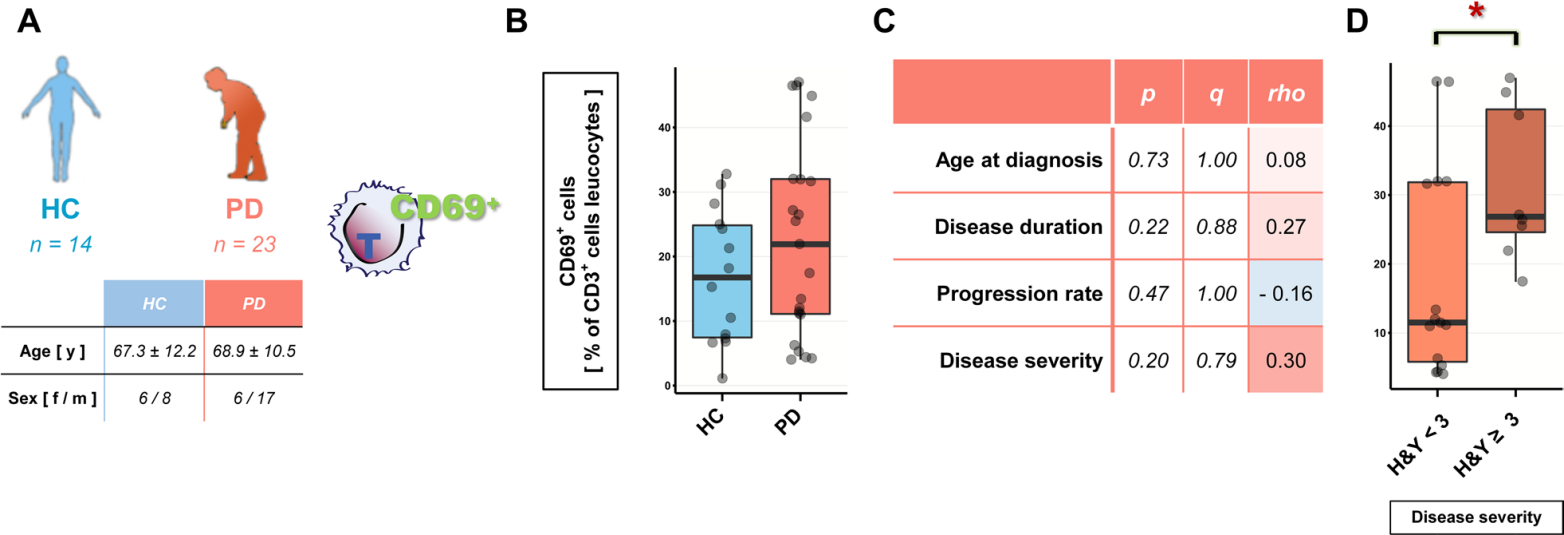
Increased activation of PD T cells by PHA is associated with disease progression. **A**, **B** CD3^+^ T cells were enriched from peripheral blood, stimulated with PHA and T-cell activation assayed by quantitation of CD69 expression on the cell surface. Activation was slightly increased in PD patients’ T cells [HC *(n* = 14, f/m = *6/8*) and PD patients *(n* = 23, 6/17 f/m), *p* < *0.09*, two-way ANOVA: case *p* < *0.10*, sex *p* > *0.99*, age *p* < *0.16*]. **C** Correlation of T-cell activation with PD clinical traits (*Spearman’s ρ*). **D** T-cell activation is significantly increased in PD patients with advanced disease progression (H&Y ≥ 3. two-way ANOVA: H&Y *p < *0.05*, sex *p* > *0.25*, age *p* > *0.51*). Boxplots: median ± interquartile range; *p < 0.05, Mann–Whitney *U* test, two-way ANOVA

To investigate the functional relevance of this finding, we next mimicked acute inflammatory response by stimulating T cells with PHA and measured T-cell activation with flow cytometry using CD69 staining in an independent cohort of 23 PD (mean age 68.9 ± 10.5; f/m = 6/17) patients and 14 healthy controls (mean age 67.3 ± 12.2; f/m = 6/8) (Fig. 4A). As with gene expression, we found only a slight, statistically non-significant increase of T-cell activation by PHA in PD patients’ T cells (Fig. 4B, *p* > 0.32). Activation of T cells was not significantly correlated to any clinical trait; however, stratification into discrete clinical traits revealed a significantly increased activation of T cells in PD patients with more advanced disease (Fig. 4D, *p* < 0.05, no statistically significant bias by age or sex). T-cell activation did not significantly differ in all other discretely stratified clinical traits.

## Discussion

In the present work, we investigated the association of the clinical parameters of PD *age at diagnosis*, *disease duration*, *disease progression rate* and *disease severity* as well as age and sex of the probands with changes of T-cell numbers, expression of inflammatory genes in T cells, and activation of T cells by PHA. Through thorough statistical analysis, we demonstrate that disease severity is the only clinical trait correlating with T-cell pathology independently from age, sex and other clinical traits of PD. Our data suggests that total CD3^+^ numbers and expression of inflammatory markers are robust markers of PD severity and warrants further validation with standardized procedures in large, multi-center approaches.

Standardized T-cell counts and phenotyping are an easily accessible, automatable readout which can be routinely utilized at relative low costs to longitudinally track disease progression in controlled settings, such as clinical trials for disease-modifying therapies. However, determining T-cell features suffers from lack of specificity; thus, it is necessary to fully understand common confounding factors and ideally to complement it by an additional readout that increases specificity. We address this paradigm by demonstrating that both CD3^+^ cell numbers and inflammatory gene expression in T cells correlate mainly with disease severity. The main limitation of our study is the relatively low cohort number, limiting the statistical power and thus the certainty with which correlation with other clinical parameters can be ruled out. However, the cohort size in this study is sufficient to demonstrate that disease severity is the main clinical trait that defines the measured T-cell features. Indeed, several other studies have found that the relative abundance of *subsets* of T cells correlates with disease severity: CD4 + T cells [15, 18], Th1/Th2 T cells [21], Th17/Treg cells [21, 26]. The relative reduction of a leucocyte subset can result from its own decrease or from an increase of the other subsets in the superset. Therefore, it is important to base all analyses on an absolute leucocyte or lymphocyte count. In our study, we chose the total number of CD3^+^ T cells as a readout, which has been verified numerous times previously by absolute counts [14–16, 18]. Further factors that could not be taken in consideration in this study, but should be included in future large-scale analyses, concern diurnal variation in leucocyte subsets, bias by comorbidities, CMV serology and effect of medication. The latter two have been partially addressed in previous studies, which found no significant bias by these factors [5, 14]. One further study demonstrated a clear effect specifically of levodopa on T-cell numbers [27], and it is also known that dopamine has a strong effect on T cells. Thus, levodopa medication may induce or enhance the T-cell decrease in PD patients. However, it is unlikely that levodopa medication confounds the association of T-cell decrease with disease severity, since the majority (88%) of the PD patients in our immunophenotyping study were treated with levodopa.

Interestingly, recent breakthrough studies have demonstrated that alpha-synuclein-specific T cells are increased in PD patients, probably in association with risk haplotypes of HLA, and suggest an autoimmune involvement of T cells in PD [8, 11]. A causal role of alpha-synuclein reactive T cells was recently reinforced also by an animal model study [36]. The occurrence of alpha-synuclein-reactive T cells was increased years before motor onset in a case study and their frequency was highest around and shortly after motor onset in a larger cross-sectional cohort of PD patients [11]. After motor onset, the T-cell response to alpha-synuclein declined with increasing disease duration. By contrast, our data suggests a progressing *generalized* T-cell dysfunction with ongoing disease, probably reflecting the combined effect of ongoing inflammation, medication and lifestyle change. Thus, specific T-cell reactivity to alpha-synuclein will be more informative *before* and *around* disease onset, e.g., in prospective clinical studies, while *generalized* T-cell dysfunction will be more appropriate for tracking disease progress in disease-modifying trials.

To our knowledge, our study is the first to address the association of T-cell gene expression and clinical parameters of PD. While the sample size of the PD group was too small for reliable correlation of single genes with disease parameters, statistical power was sufficient for the blockwise correlation of gene expression with disease parameters. Indeed, we found a correlation of disease severity with modules of inflammatory gene expression, which could be validated also on a functional level. Further studies with larger numbers of PD patients are warranted to select optimal gene targets for expression analysis to complement T-cell counts for increased specificity of disease severity tracking.

## Data Availability

The data sets analysed during the current study are available from the corresponding author on reasonable request.

## Abbreviations

PD: Parkinson’s Disease
CNS: Central nervous system
HC: Healthy control
HY: Hoehn & Yahr stage
PBMC: Peripheral blood mononuclear cell
GO: Gene Ontology
RT-qPCR: Quantitative reverse-transcription PCR
PCR: Polymerase chain reaction
ANOVA: Analysis of variance
FDR: False discovery rate
PHA: Phytohemagglutinin
